# Respiratory tract isolation of *Mycobacterium europaeum* following influenza infection in an immunocompromised patient: a case report

**DOI:** 10.1186/1752-1947-8-463

**Published:** 2014-12-25

**Authors:** Michael Phelippeau, Marion Delord, Michel Drancourt, Philippe Brouqui

**Affiliations:** 21 Aix Marseille Université, URMITE, UM63, CNRS 7278, IRD 198, Inserm 1095, Institut Hospitalo-Universitaire « Méditerranée Infection », AP-HM, 13005 Marseille, France; 22 Unité de recherche sur les maladies infectieuses et tropicales émergentes, Faculté de Médecine, 27 Bd jean Moulin, 13385 Marseille, CEDEX 5, France

**Keywords:** HIV, Influenza, MALDI-TOF-MS, *Mycobacterium europaeum*, *rpoB*

## Abstract

**Introduction:**

*Mycobacterium europaeum*, a slow-growing nontuberculous mycobacteria belonging to the *Mycobacterium simiae* complex, was described after the seminal characterization of five isolates collected from three sputum specimens and a jaw gland biopsy in Italy, Greece and Sweden. Five respiratory tract isolates were further reported in Iran. Here, we report the first isolation of *M. europaeum* in France, in the respiratory tract of a patient co-infected with human immunodeficiency virus and hepatitis C virus.

**Case presentation:**

A 49-year-old Caucasian woman with a 26-year history of human immunodeficiency virus-hepatitis C virus co-infection was admitted for significant influenza-like syndrome in a context of repetitive exacerbations of chronic obstructive pulmonary disease. Significant biological parameters included lymphocytes of 1.6G/L including 237/mm^3^ T4 lymphocytes, a human immunodeficiency virus viral load of 1.6 log and a hepatitis C virus viral load of 6 log. Reverse-transcriptase polymerase chain reaction of her nasopharyngeal aspiration confirmed influenza A H1N1. Three sputum specimens lacked acid-fast bacilli but one grew mycobacteria identified by using matrix-assisted laser desorption ionization/time-of-flight mass spectrometry as *M. europaeum* with a 1.56 log score. A 1,482-bp 16S ribosomal ribonucleic acid gene sequence yielded 99% similarity with both *Mycobacterium parascrofulaceum* ATCC BAA-614 and *M. europaeum* DSM 45397^T^ and partial *rpoB* polymerase chain reaction-sequencing yielded a 725-bp sequence exhibiting 100% similarity with *M. europaeum* strain DSM 45397^T^.

**Conclusions:**

We report the first isolation of *M. europaeum* in France, in the respiratory tract of a patient co-infected with human immunodeficiency virus and hepatitis C virus. *M. europaeum* warrants further attention in immunosuppressed patients with influenza, using matrix-assisted laser desorption ionization/time-of-flight mass spectrometry and *rpoB* partial sequencing as tools for its accurate identification.

## Introduction

*Mycobacterium europaeum*, a slow-growing nontuberculous mycobacteria (NTM) belonging to the *Mycobacterium simiae* complex, was described after the seminal characterization of five isolates collected from three sputum specimens and a jaw gland biopsy in Italy, Greece and Sweden [1]. Five respiratory tract isolates were further reported in Iran [2] from two different infected patients according to the American Thoracic Society/Infectious Diseases Society of America (ATS/IDSA criteria for NTM lung disease [3]. In fact, ATS/IDSA criteria for NTM lung infection combine clinical pulmonary criteria, new radiological opacities, and, positive culture results from several respiratory tract samples [3]. Here, we report the first isolation of *M. europaeum* in France, in the respiratory tract of a patient co-infected with human immunodeficiency virus (HIV) and hepatitis C virus (HCV).

## Case presentation

**Figure 1:**
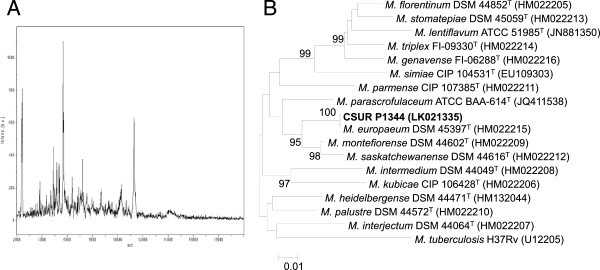
**Matrix-assisted laser desorption ionization/ time-of-flight analysis of** ***Mycobacterium europaeum*** **. A**. Matrix-assisted laser desorption ionization/time-of-flight mass spectrometry profile of *Mycobacterium europaeum* CSUR P1344 using colonies from egg-based Löwenstein–Jensen medium. **B**. Phylogenetic tree constructed using the neighbor-joining method (bootstrapped 1000 times) and Kimura’s two-parameter distance correction model based on *rpoB* partial sequences of 18 mycobacterial species including isolate CSUR P1344 collected in our patient’s sputum. Bootstrap values above 90% are given at nodes. *Mycobacterium tuberculosis* H37Rv was used as out-group. The scale bar represents 1% difference in nucleotide sequences. GenBank accession numbers are given in parentheses. *M*. is *Mycobacterium.*

A 49-year-old Caucasian woman with a 26-year history of HIV-HCV co-infection was admitted to an Infectious and Tropical Diseases Department in Marseille, France in February 2014 for significant influenza-like syndrome in a context of repetitive exacerbations of chronic obstructive pulmonary disease. Pulmonary auscultation found diffuse wheezing. Significant biological parameters included a C-reactive protein level of 57mg/L, lymphocytes of 1.6G/L including 237/mm^3^ T4 lymphocytes, a HIV viral load of 1.6 log and a HCV viral load of 6 log. Her chest radiography was normal. Reverse-transcriptase polymerase chain reaction (PCR) of her nasopharyngeal aspiration confirmed influenza A H1N1. At a 6-month follow-up, her clinical outcome was favorable with initial supportive treatment only. Three sputum specimens lacked acid-fast bacilli (AFB) after Ziehl–Neelsen staining and microscopic observation but one grew AFB after 21-day incubation in MGIT (Becton Dickinson, Le Pont-de-Claix, France) at 37°C in a 5% carbon dioxide atmosphere. After subculture on Coletsos (bioMérieux, La-Balme-les-Grottes, France), the isolate was deposited in our collection (CSUR P1344) and tentatively identified by using matrix-assisted laser desorption ionization/time-of-flight mass spectrometry (MALDI-TOF-MS), the bioMérieux extraction protocol, a MicroFlex™ mass spectrometer (Bruker Daltonics, Bremen, Germany) and the Bruker MALDI Biotyper procedures as previously described [4]. A reproducible profile (Figure 1A) yielded a 1.56 log score with *M. europaeum* DSM 45397^T^ listed in the Bruker Mycobacterium Library 1.0 and 2.0 databases. A 1,482-bp 16S ribosomal ribonucleic acid (rRNA) gene sequence (GenBank LN680852) yielded 99% similarity with both *Mycobacterium parascrofulaceum* ATCC BAA-614 (GenBank GQ153273) and *M. europaeum* DSM 45397^T^ (GenBank HM022196), two species known to share almost identical 16S rRNA gene sequence [1]. Partial *rpoB* PCR-sequencing [5] yielded a 725-bp sequence (GenBank LK021335) exhibiting 100% similarity with *M. europaeum* strain DSM 45397^T^ (GenBank HM022215; Figure 1B).

Here, one *M. europaeum* isolate was made from broth culture whereas the eight previous isolates have been made from agar or egg-based solid medium [1, 2], illustrating the capability of automatons routinely used in laboratories, to detect the growth of *M. europaeum*. Starting from colonies, this isolate was identified by MALDI-TOF-MS and its profile made freely available at Méditerranée Infection Foundation URMS Database [6] in order to complement the sole profile (*M. europaeum* DSM 45397^T^) commercially available.

## Conclusions

*M. europaeum* has not been previously reported in France. Three of the five seminal strains have been isolated from sputum. Of interest, two Iranian patients [2] with a history of acquired immunodeficiency syndrome and cystic fibrosis fulfilled the ATS/IDSA criteria for NTM pulmonary infection [3]. Here, clinical, radiological and microbiological findings classified this case as a colonized case [3]. As our understanding about the pathophysiology of *M. europaeum* lung infection is insufficient, there is not enough known to be sure that colonization is not in fact a slowly progressive infection. In addition, like other species of the *M. simiae* complex [7, 8], this new species could be clinically relevant when isolated in the respiratory tract of immunocompromised hosts. In fact, NTM should be investigated in the respiratory tract samples of immunocompromised patients because of a higher risk of lung infection that needs particular treatment [3]. Of interest, *M. europaeum* was here isolated in a patient with laboratory-confirmed influenza, a condition previously associated with *Mycobacterium tuberculosis* infection [9] through virus-induced interference in the interferon pathway [10]. Interactions between influenza and superinfecting mycobacteria, particularly NTM in the respiratory tract, warrant further attention to be elucidated.

## Consent

Written informed consent was obtained from the patient for publication of this case report and any accompanying images. A copy of the written consent is available for review by the Editor-in-Chief of this journal.
